# From Stool to Scope: Optimising FIT Thresholds to Guide Future Panenteric Capsule Endoscopy and Reduce Colonoscopy Burden in Iron Deficiency Anaemia

**DOI:** 10.3390/cancers17121951

**Published:** 2025-06-11

**Authors:** Ian Io Lei, Nicola O’Connell, Michael Agyekum Adu-Darko, Jessiya Parambil, Vishnupriya Suresh, Kiara Mc Donnell, Jessie Newville, Kirsten Chaplin, Deekshi Siyambalapityage, Asad Khan, Usman Muhammad, John Emil, Merali Abbas, Zia Kanji, Omar Khalil, Hamza Alam, Amelia Bennett, Hannah Soanes, Adrija Bhattacharyya, Karl Frey, Rosie Meakins, Archit Singhal, George Pack, Melike Gerrits, Harry Paterson, Vincent Cheung, Sue Cullen, Imran Aslam, Chander Shekhar, Ramesh P. Arasaradnam

**Affiliations:** 1 Institute of Precision Diagnostics & Translational Medicine, University Hospital of Coventry and Warwickshire, Clifford Bridge Rd, Coventry CV2 2DX, UK; nicola.oconnell@uhcw.nhs.uk (N.O.); vishnupriya.suresh@uhcw.nhs.uk (V.S.); omar.khalil@uhcw.nhs.uk (O.K.); imran.aslam@uhcw.nhs.uk (I.A.); chander.shekhar@uhcw.nhs.uk (C.S.); r.arasaradnam@warwick.ac.uk (R.P.A.); 2 Warwick Medical School, University of Warwick, Coventry CV4 7AL, UK; 3 Department of Gastroenterology, Milton Keynes University Hospital, Standing Wy, Eaglestone, Milton Keynes MK6 5LD, UK; michael.adu-darko@mkuh.nhs.uk (M.A.A.-D.); asad.khan@mkuh.nhs.uk (A.K.); muhammad.usman@mkuh.nhs.uk (U.M.); emil.john@mkuh.nhs.uk (J.E.); abbas.merali@mkuh.nhs.uk (M.A.); zia.kanji@mkuh.nhs.uk (Z.K.); hamza.alam3002@gmail.com (H.A.); 4 Department of Gastroenterology, Oxford University Hospitals NHS Foundation Trust, John Radcliffe Hospital, Headley Way, Headington OX3 9DU, UK; jessiya.parambil@ouh.nhs.uk (J.P.); amelia.bennett@ouh.nhs.uk (A.B.); hannah.soanes@ouh.nhs.uk (H.S.); adrija.bhattacharyya@ouh.nhs.uk (A.B.); karl.frey@ouh.nhs.uk (K.F.); rosie.meakins@ouh.nhs.uk (R.M.); archit.singhal@ouh.nhs.uk (A.S.); georgepack2@gmail.com (G.P.); melike.gerrits@ouh.nhs.uk (M.G.); harry.paterson@ouh.nhs.uk (H.P.); vincent.cheung@ouh.nhs.uk (V.C.); 5 Department of Gastroenterology, Stoke Mandeville Hospital, Buckinghamshire Healthcare NHS Trust, Mandeville Rd, Aylesbury HP21 8AL, UK; kiara.mcdonnell@nhs.net (K.M.D.); jessie.newville@nhs.net (J.N.); kirsten.chaplin1@nhs.net (K.C.); deekshi.siyambalapitiyage@nhs.net (D.S.); sue.cullen1@nhs.net (S.C.); 6 Leicester Cancer Centre, University of Leicester, Leicester LE1 7RH, UK

**Keywords:** colon capsule endoscopy, panenteric capsule endoscopy, colonoscopy, capsule endoscopy, iron deficiency anaemia, anaemia, IDA, colorectal cancer, polyp

## Abstract

**Background:** Colon capsule endoscopy (CCE) or panenteric capsule endoscopy (PCE) offers a promising, non-invasive diagnostic approach for patients with iron deficiency anaemia (IDA). However, high rates of conversion to conventional colonoscopy (CCC) following capsule procedures reduce cost-effectiveness and patient satisfaction. Optimising the faecal immunochemical test (FIT) threshold may improve patient stratification and reduce unnecessary conversions in future applications within the IDA diagnostic pathway. **Methods:** The CLEAR IDA study was a multicentre, retrospective observational study conducted across four UK hospitals. Data were collected over a six-month study period and included patients referred via the two-week-wait (2WW) cancer pathway for iron deficiency, with or without anaemia, over a 12-month timeframe. Colonoscopy findings were analysed and extrapolated using NHS England’s CCE-to-colonoscopy referral criteria to assess the predictive value of FIT for colorectal cancer (CRC), polyp burden, and CCC using ROC curve analysis. The optimal FIT threshold was identified through three complementary approaches: threshold-based analysis, decision curve analysis, and cost–benefit modelling. **Results:** A total of 1531 patients were analysed; only 1.6% underwent small bowel capsule endoscopy. The diagnostic accuracy (AUC) of FIT for predicting CRC, polypoidal lesions, and CCC was 0.78, 0.58, and 0.69, respectively. Threshold-based analysis identified FIT = 15 µg/g as the lowest level at which CCC rates significantly increased (*p* = 0.02; OR = 1.87; 95% CI: 1.07–3.14). Decision curve analysis showed a maximum net benefit at FIT = 17.6 µg/g, while cost–benefit modelling identified 9 µg/g as the most cost-effective. Raising the threshold to 10 µg/g resulted in a net loss of GBP –294.4 per patient. An optimal cost-effective FIT threshold range was identified between 10 and 17.6 µg/g. The threshold selection should be tailored to local service capacity and resource availability. **Conclusions:** While FIT alone is an imperfect triage tool, optimising thresholds between 10 and 17 µg/g may enhance cost-effectiveness and guide appropriate PCE use in IDA.

## Simple Summary

This study looked at how to make better use of panenteric capsule endoscopy—a small camera that is swallowed to examine both small and large bowels—for investigating iron deficiency anaemia (IDA). This condition can signal bowel problems like cancer. Many patients who have a regular colonoscopy, which can be uncomfortable and costly, still end up needing a capsule test to further examine the small bowel. Researchers studied over 1500 patients in four UK hospitals to see if adjusting the cutoff level for a stool test called FIT could aid in triaging a single capsule test that examines both small and large bowels. FIT checks for hidden blood in the stool and is often used to decide who needs further colonoscopies. The study found that setting the FIT level between 10 and 17.6 µg/g could reduce unnecessary colonoscopies by offering a panenteric capsule test to low-risk patients, saving costs without missing important findings. Although FIT is not perfect on its own, choosing the right cutoff can improve how doctors decide who truly requires more invasive tests. The best level may depend on local NHS resources.

## 1. Introduction

Iron deficiency anaemia (IDA) is a common indication for full gastrointestinal (GI) evaluation, primarily to rule out malignancy-related blood loss. However, the diagnostic yield of significant upper or lower GI pathology, particularly gastric and colorectal cancer, remains low, often resulting in repeated OGDs and colonoscopies before small bowel assessment is considered [1,2,3]. The British Society of Gastroenterology (BSG) recommends small bowel investigations for patients with recurrent IDA or inadequate response to iron therapy [4]. Recent evidence has shown that capsule endoscopy has a high diagnostic yield in patients with occult GI bleeding, supporting its potential use as a first-line investigation in this setting [5]. This has prompted interest in using panenteric capsule endoscopy (PCE) as an initial diagnostic tool to save time, reduce healthcare costs, and potentially lessen environmental impacts. In recent years, colon capsule endoscopy (CCE) has gained traction in the UK as a less invasive alternative for lower gastrointestinal evaluation [6,7]. Although colon capsule endoscopy (CCE) is primarily licensed for colonic evaluation, it can be extended to PCE by simply deactivating its sleep mode, enabling visualisation of the entire gastrointestinal tract. This functionality closely mirrors that of the PillCam™ Crohn’s capsule, the device formally licensed for panenteric use, with both systems sharing similar technical specifications and capsule delivery protocols.

Building on BSG guidance and the expanded functionality of capsule technology, PCE presents a logical first-line investigation strategy for iron deficiency anaemia (IDA). However, concerns remain regarding its cost-effectiveness. Insights from large-scale studies such as SCOTCAP highlight this issue: 59% of patients required follow-up conventional endoscopy after initial colon capsule endoscopy (CCE), due to factors such as poor bowel preparation, incomplete examinations, or findings requiring biopsy or therapeutic intervention (see Table S1 in the Supplementary Materials) [8,9]. These CCE-to-colonoscopy conversions (CCCs) not only undermine cost-efficiency but also increase patient burden related to repeat bowel preparation. A promising solution is the incorporation of quantitative faecal immunochemical testing (qFIT) to stratify CCC risk. qFIT is already widely used in colorectal cancer (CRC) screening programmes and as a triage tool for CCE referrals [6,7]. It has demonstrated strong performance in detecting CRC and advanced neoplasia across various thresholds [1]. In SCOTCAP, qFIT values between 10 and 399 μg/g were associated with higher rates of further investigation, while NHS England’s use of an arbitrary cutoff of ≤100 μg/g led to a reduced 49% follow-up endoscopy rate [7]. However, the absence of robust cost-effectiveness modelling across different thresholds limits the optimal integration of qFIT into CCE or PCE clinical pathways.

This multicentre retrospective study aims to evaluate the accuracy of faecal immunochemical testing (FIT) in predicting colorectal cancer (CRC) and polyp burden in patients with iron deficiency anaemia (IDA). Given the diagnostic comparability between CCE and colonoscopy for polyp detection [10,11], colonoscopy findings will be analysed and extrapolated using NHS England’s CCE-to-colonoscopy referral criteria. The goal is to identify an optimal FIT threshold to support the use of CCE or panenteric capsule endoscopy (PCE) as a cost-effective first-line investigation for IDA [12]. Ultimately, this approach aims to reduce unnecessary CCC, enhance diagnostic efficiency, and streamline the IDA cancer referral pathway in the future.

## 2. Methods

### 2.1. Study Design and Participants

The **C**CE **E**ndoscopic **A**ssessment and **R**eferral using qFIT in **I**ron **D**eficiency **A**naemia (CLEAR IDA) study was a multicentre, retrospective observational study involving the collection of patient data spanning a 12-month timeframe from 1 September 2023 to 1 September 2024. It included patients referred via the two-week-wait cancer (2WW) pathway for iron deficiency with or without anaemia, irrespective of symptoms. IDA was defined according to BSG guidelines: anaemia was classified as haemoglobin below the local lower limit of normal, while iron deficiency was indicated by a low mean corpuscular volume (MCV), low ferritin, or a combination of low iron and transferrin saturation [4]. Eligibility criteria included patients aged 18 years or older who underwent both gastroscopy and either colonoscopy or CT virtual colonoscopy within the study period. Small bowel capsule endoscopy data were also recorded when available. The exclusion criteria comprised patients with iron deficiency anaemia (IDA) who were deemed unfit for lower gastrointestinal investigations, as this group is unlikely to benefit from PCE or CCE [13]. Patients with non-iron deficiency or normocytic anaemia, including those presenting with acute gastrointestinal bleeding, were excluded. Patients who had opted out of national audit data collection for research purposes were also excluded. In the UK, audit data collection operates on a default opt-in basis, whereby non-exclusion implies consent. All included patients were locally identified and screened retrospectively at participating hospitals from their two-week-wait cancer referral and standard endoscopic workup pathways.

This study was conducted across two secondary care hospitals and two tertiary teaching hospitals in the United Kingdom from seven invited sites. Recruitment and coordination were facilitated through trainee-led collaborative networks between regional deaneries. Data collection was carried out using a pre-designed Microsoft Excel template completed by local clinical teams. Bowel preparation regimens and endoscopic procedures followed each site’s established clinical protocols. All participating centres were accredited by the Joint Advisory Group (JAG) as part of the UK’s endoscopy quality assurance and key performance indicator (KPI) framework [14]. The data were directly collected from electronic patient records, laboratory results, and endoscopy and radiology reports.

### 2.2. Outcome Measures

The primary aim of this study was to evaluate the accuracy of faecal immunochemical testing (FIT) in predicting colorectal cancer (CRC) and polyp burden in patients with iron deficiency anaemia (IDA), and to extrapolate these findings to assess FIT’s utility in predicting the likelihood of conversion from CCE to conventional colonoscopy (CCC) based on an underlying pathology in IDA. As a secondary outcome, this study explored other contributory factors that may influence the likelihood of CCC.

### 2.3. Statistical Analysis

A sample size calculation was performed to ensure sufficient power to evaluate the predictive accuracy of faecal immunochemical testing (FIT) in identifying clinically significant colonic pathology in patients with iron deficiency anaemia (IDA) to predict CCC. Using prior assumptions of an expected difference of 20 μg/g in FIT values between patients with and without significant findings and a standard deviation of 117 μg/g, an effect size of 0.8 and a Cohen’s d of 0.17 were estimated from a previous study [15]. Applying a two-sided alpha of 0.05 and 90% statistical power, the required sample size was calculated to be 1441 patients (see study protocol in the Supplementary Materials).

Patient demographic and clinical characteristics were summarised using descriptive statistics. Continuous variables were reported as mean ± standard deviation (SD). Although patients with and without recorded FIT values were initially considered during the screening phase, only those with complete data—including a valid FIT result, documented number of polyps, and measurement of the largest polyp size—were included in the final analysis. For analysis, FIT values reported as <7 μg/g, <10 μg/g, and >400 μg/g were imputed as exact values of 7, 10 and 400 μg/g, respectively, to enable statistical modelling. The predictive accuracy of FIT for colorectal cancer (CRC), polypoidal lesions, and the likelihood of conversion to conventional colonoscopy (CCC) was assessed using Receiver Operating Characteristic (ROC) curve analysis. The area under the ROC curve (AUROC) was calculated both unadjusted and adjusted for potential confounding factors to evaluate the independent discriminative performance of FIT. The primary analyses were conducted on a complete-case basis, including only participants with no missing data for the variables of interest. Demographic variables with substantial amounts of missing data were excluded from the analysis. All available predictors were assessed using univariate and multivariate logistic regression models. The results are presented as odds ratios (ORs) with corresponding 95% confidence intervals (CIs).

To evaluate the clinical utility of FIT as a triage tool for urgent colonoscopy, three complementary analytic approaches were employed:

(1) A threshold-based analysis was conducted using the Cumulative Sum Control (CUSUM) Charts by incrementally increasing FIT values (0.1 µg/g) and calculating the cumulative number of urgent colonoscopies at each level. Sharp increases (≥5 cases) were identified as “jump points”, indicating potential inflection thresholds. Corresponding conversion rates were calculated as the proportion of urgent cases at or below each FIT threshold [16].
(2) A cost–benefit analysis was performed to estimate the net benefit per patient across varying FIT thresholds, incorporating the trade-offs between true positives, true negatives, false positives, and false negatives using predefined clinical cost and outcome values (see Table S2 in the Supplementary Materials) [17,18].
(3) A decision curve analysis (DCA) was conducted using a logistic regression model, with the binary outcome variable “Urgent Colonoscopy” modelled as a function of continuous FIT values. Predicted probabilities were used to calculate the net benefit across threshold probabilities ranging from 0 to 1 (in 0.01 increments), classifying patients as high or low risk based on each threshold [19,20,21,22].

The R packages used for data analysis and graph creation included “pwr” [23], “dplyr” [24], “pROC” [25], “ggplot2” [26], and “broom” [27]. A significance level of 0.05 was used for all analyses, and modelling was conducted using R software, version 2024.12.1 [28]. The R code used for this analysis is available upon request.

### 2.4. Ethical Approval

The CLEAR IDA study was conducted as an audit and service evaluation project; therefore, approval from a research ethics committee was not required. The evaluation was carried out in accordance with the UK NHS Health Research Authority and Medical Research Council decision-making tool for clinical audit and service evaluation [29]. The project was formally registered as an audit or service evaluation at each participating hospital, with prospective approval obtained prior to data collection.

## 3. Results

**Figure 1:**
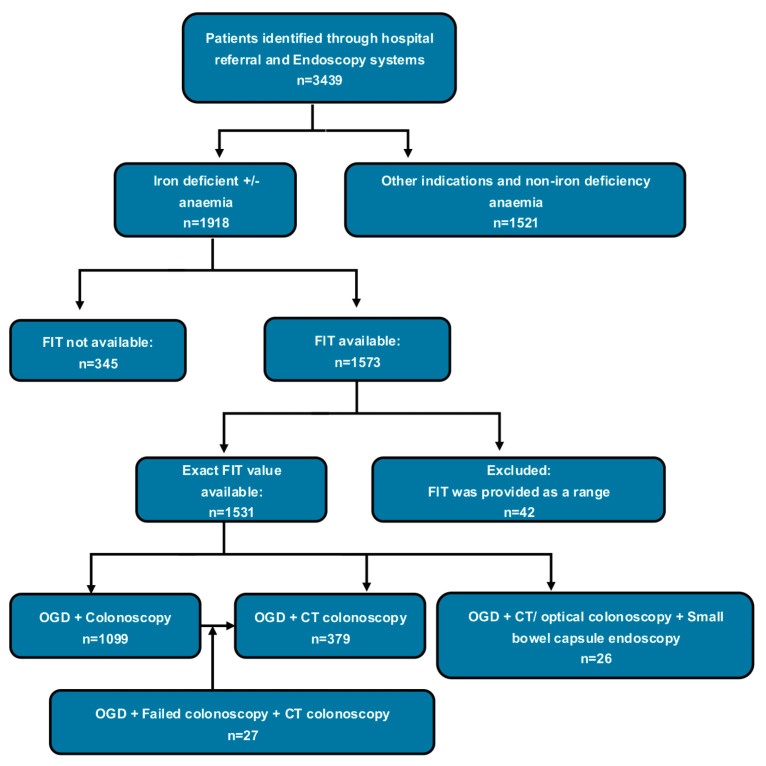
Flow diagram showing the number of patients who had both upper and lower investigations with available FIT test.

A total of 1531 patients who had both upper and lower investigations and available FIT results were included in the analysis (see flow diagram Figure 1). Eighteen per cent of iron deficiency anaemia patients did not undergo a FIT test. Table 1 shows the participants’ demographics and characteristics. An amount of 71.8% of patients had colonoscopy while 24.8 had CT colonoscopy; 1.8% of patients had an incomplete colonoscopy and required further CT colonoscopy; 1.6% had small bowel capsule endoscopy after the initial upper and lower GI endoscopic investigation; 13.8% of patients had advanced polyps (size ≥ 10 mm), within which 6.3% were CRC. The patient distribution curve by FIT level showed that the majority of patients had FIT values below 30 µg/g (see Figure S1, Supplementary Materials).

### 3.1. The Accuracy of FIT in Predicting CRC, Polyp, and CCC (Urgent)

**Figure 2:**
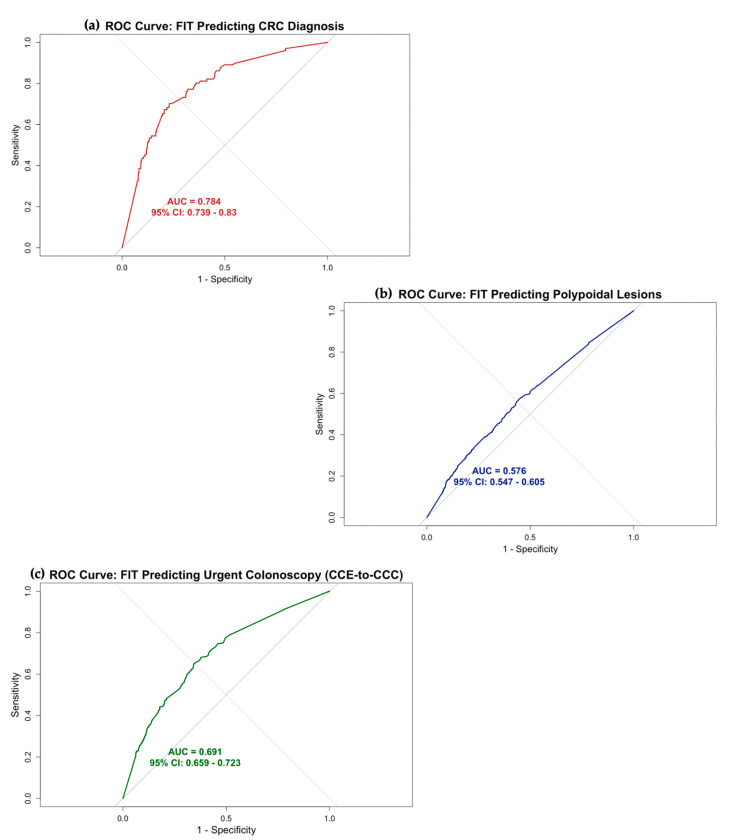
Receiver Operating Characteristic (ROC) curves showing (**a**) FIT performance in predicting colorectal cancer (CRC), (**b**) FIT performance in predicting polypoid lesions (including both polyps and CRC) and (**c**) FIT performance in predicting CCE to urgent colonoscopy conversion (CCC). The area under the curve (AUC) was calculated for each ROC curve to assess the diagnostic accuracy of FIT in detecting these pathologies within the IDA cohort.

The diagnostic accuracy of FIT in predicting colorectal cancer (CRC), polypoidal lesions, and conversion to conventional colonoscopy (CCC) yielded AUCs of 0.784 (95%CI: 0.739–0.830), 0.576 (95%CI: 0.547–0.605), and 0.691 (95%CI: 0.659–0.723), respectively. These results illustrated in the ROC curves in Figure 2 highlight the limited predictive power of FIT for polyp detection and, by extension, its ability to predict CCC. Univariate and multivariate logistic regression analyses are presented in Table S3 (Supplementary Materials). Although all four variables—FIT, haemoglobin, age, and sex—were statistically significant predictors of CCC, the multivariable model did not improve overall diagnostic performance compared to the FIT-only model, as demonstrated in the ROC comparison shown in Figure S2 (Supplementary Materials).

### 3.2. Threshold-Based Analysis

**Figure 3:**
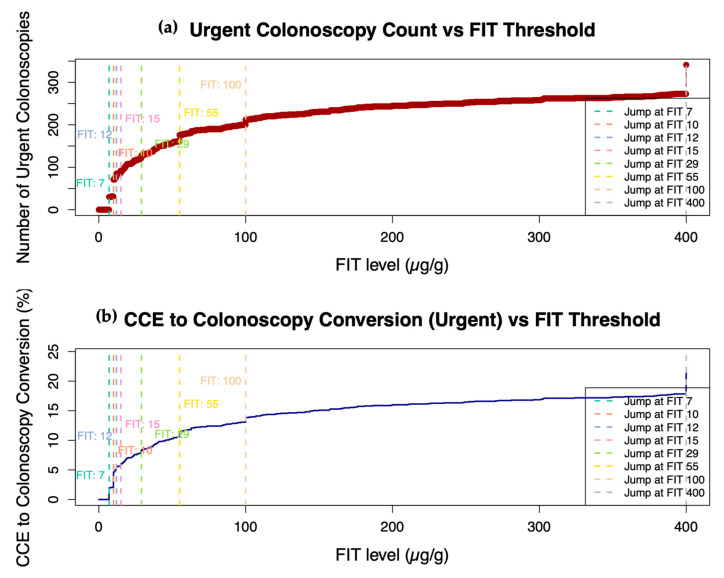
Cumulative Sum Control (CUSUM) charts extrapolating (colonoscopy) colonic pathology findings to predict CCE to urgent colonoscopy conversions (Urgent CCC). Chart (**a**): Number of urgent colonoscopies required at or below each FIT threshold. Chart (**b**): Corresponding CCE-to-colonoscopy conversion rate (%) across increasing FIT thresholds. Threshold jump points, representing sharp increases in urgent colonoscopy conversions, are identified and listed in the legend on the right side of the graph.

Cumulative Sum Control (CUSUM) charts are shown in Figure 3, highlighting dynamic increases—defined as jumps of ≥5 cases—in the number of urgent colonoscopies across incremental FIT thresholds. Eight FIT thresholds were identified as inflection points: 7, 10, 12, 15, 29, 55, 100, and 400 µg/g. Notably, the jumps observed at FIT levels 7, 10, and 400 reflect the lower and upper detection limits of FIT assays used at different sites. The corresponding number of urgent colonoscopies, colon capsule to colonoscopy conversion (CCC) rates, and absolute rate differences for each jump threshold are detailed in Table S4 (Supplementary Materials). A more pronounced increase in the CCC conversion rate was observed beyond FIT = 15, rising from 0.5% at FIT = 15 to 2.4% at FIT = 29, with further notable increases seen at all higher FIT thresholds thereafter.

To further refine the optimal FIT threshold, particularly within the 10–29 µg/g range identified in the threshold-based analysis, we investigated the point (FIT level) and the likelihood of conversion became statistically significant (see Figure S4 in the Supplementary Saterials). Patients were categorised into two groups: those with FIT values ≤10 µg/g (Group 1, low FIT) and those with values between 11 and 29 µg/g (Group 2, rising FIT). Using logistic regression, we compared the likelihood of urgent referral between these groups at each incremental FIT value within this range. This approach enabled us to determine the lowest FIT threshold at which the increase in CCC reached statistical significance (*p* < 0.05) persistently. A visual plot illustrating the evolution of statistical significance across the FIT range is presented in Figure S3 (Supplementary Materials), helping to identify a clinically meaningful decision point for patient triage. The analysis revealed that a sustained and statistically significant increase in CCC was only observed beyond a FIT level of 15 µg/g. Using a cutoff of 15 µg/g, the subgroup analysis showed a statistically significant difference compared to the FIT ≤10 µg/g group, with a *p*-value of 0.02 and an odds ratio (OR) of 1.87 (95% CI: 1.07–3.14), as illustrated in Figure S3 (Supplementary Materials).

### 3.3. Decision Curve and Cost–Benefit Analysis for FIT Thresholds to Minimise CCC

**Figure 4:**
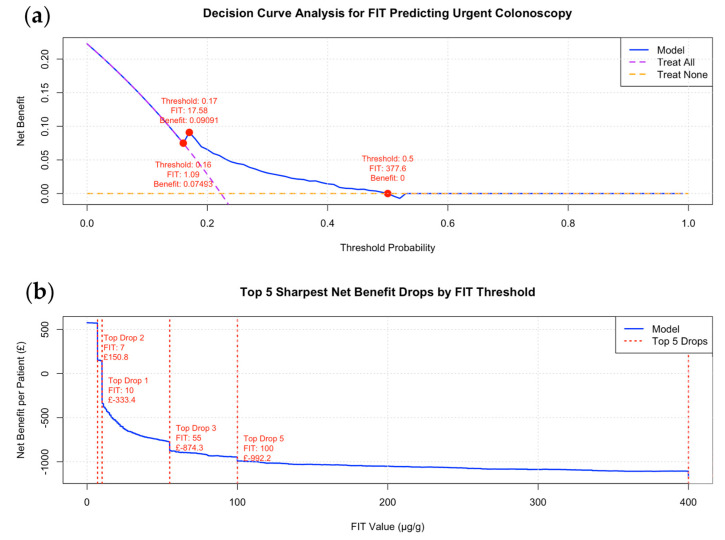
(**a**) Decision curve analysis (DCA) illustrating net benefit for FIT in predicting urgent colonoscopy. Three key threshold probabilities are annotated in red, along with their corresponding FIT values and estimated net benefit. (**b**) Cost–benefit (adapted DCA) analysis showing the net monetary benefit per patient across FIT thresholds, aimed at optimising cost-effective triage in patients with iron deficiency anaemia (IDA). Red dashed lines indicate the top five threshold points associated with the steepest declines in net benefit, alongside the monetary loss (GBP) observed at each point.

A decision curve and cost–benefit analysis were conducted to evaluate the trade-offs between false-positive and false-negative predictions. In this analysis, false positives are associated with a minor financial loss due to the lower cost of colon capsule endoscopy (CCE) compared to conventional colonoscopy, whereas false negatives incur higher costs because they represent the combined expense of both procedures when CCE subsequently converts to urgent colonoscopy. These costs were weighed against the savings from true positives, who bypass the cost of an unnecessary CCE, and true negatives, who avoid the expense of undergoing a subsequent colonoscopy. Figure 4 illustrates this analysis through decision and cost–benefit curves. In this simplified model, local cost assumptions were employed, with CCE assumed to be 17% less expensive than colonoscopy, based on published costs from the SCOTCAP study (Table S2 Supplementary Materials) [9].

In the decision curve analysis, the optimal threshold range was determined by the range where the model’s curve was positioned above both the “treat all” and “treat none” lines. The optimal cost-effective predicted probability threshold was identified as falling between 0.16 and 0.5, corresponding to a FIT range of 1–377.6 μg/g but with a maximum net benefit at FIT = 17.58 μg/g (Figure 4a). The cost–benefit analysis identified 9 μg/g as the most cost-effective FIT threshold, yielding a net benefit ranging from GBP 0 to 147. However, increasing the threshold to 10 μg/g resulted in the net benefit dropping from 0 to GBP –294.4 per patient (Figure 4b). Collectively, the results from the above analyses suggest that employing a low FIT threshold (FIT = 10–17.6 μg/g) is essential to minimise CCC and the cost of the service in patients with IDA.

## 4. Discussion

This is the first study to evaluate the accuracy of FIT to predict CCC based on identified colonic pathologies and to define an optimal FIT threshold in patients with iron deficiency undergoing panenteric capsule endoscopy. The use of a panenteric capsule endoscopy can achieve a one-stop full GI tract visualisation in combination with OGD. However, like CCE, it remains limited by a high reinvestigation rate and concerns regarding the cost-effectiveness of the service.

CCE-to-colonoscopy conversion (CCC) is primarily driven by polyp size and number within the colon [15]. In our analysis, the ROC curve demonstrated that FIT had an area under the curve (AUC) of approximately 0.78 for detecting colorectal cancer (CRC), consistent with previously published data [30]. However, the AUC for detecting polypoid lesions (i.e., polyps and CRC combined) was only 0.58, reflecting poor discriminatory performance, again aligning with findings in the literature [31]. Although FIT performs relatively well in detecting CRC, it has clear limitations in identifying other pathologies, such as IBD, as demonstrated by Turvill et al. [32]. Also, some advanced polyps and malignancies may not bleed and therefore remain undetectable by FIT, reducing its utility as a standalone triage tool [33,34]. This diagnostic gap has opened new avenues for integrating adjunctive tools, such as volatile organic compound (VOC) analysis. Chandrapalan et al. reported favourable results, with VOC testing achieving a high sensitivity of 0.94 (95% CI: 0.88–0.98) for detecting advanced polyps or ≥5 polyps, though with a moderate specificity of 0.69 (95% CI: 0.64–0.75) [35]. Concurrently, recent advancements in artificial intelligence (AI) have further enhanced both the diagnostic potential and efficiency of capsule endoscopy [36,37,38]. In the context of colon capsule endoscopy (CCE), reported sensitivities for polyp detection using AI have ranged from 47.4% to 89.1%. Interim findings from the CESCAIL study have also demonstrated comparable diagnostic accuracy, alongside a significant reduction in clinician reading time, although full results are still pending [39,40].

Although FIT is not a perfect triaging tool, optimising patient selection for CCE based on FIT thresholds can help reduce CCC rates [7,12]. Having said that, the AUC for using FIT to predict CCC based on the colonic pathology was only 0.69. Although this value is higher than that previously reported by Lei et al. [15], it is important to note that our AUC was based solely on colonic pathology and did not account for other conversion drivers, such as inadequate bowel preparation or incomplete procedures. When these factors are considered, the predictive accuracy of FIT is likely to be lower.

**Figure 5:**
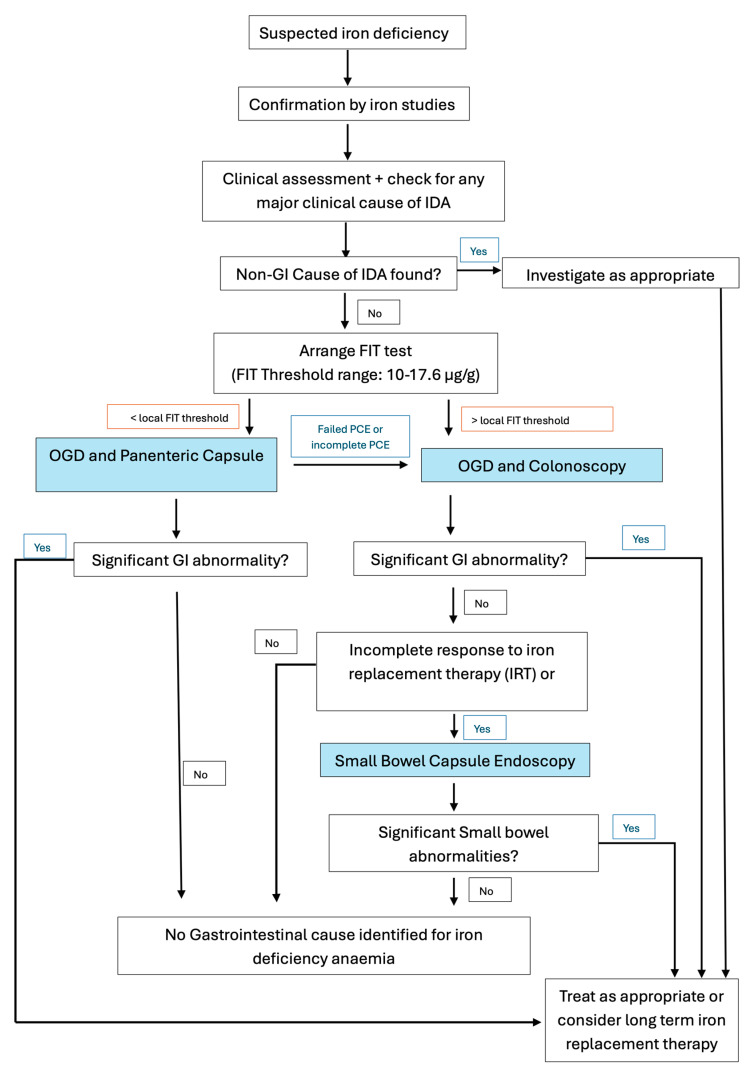
Illustration of the proposed Capsule-Assisted Pathway for Stratifying the Iron Deficiency Anaemia Pathway (CAPS-IDA Pathway), incorporating PCE for patients with IDA.

The optimal FIT threshold range of 10–17.6 µg/g was identified through three complementary analyses. When adopting FIT as a triage tool for CCE, threshold selection should be tailored to local circumstances rather than applying a universal standard. The optimal balance between diagnostic accuracy and cost-effectiveness depends on several contextual factors, particularly the size of the regional colonoscopy waiting list and the available endoscopy unit capacity. As waiting times increase and procedures are delayed, the cost-effectiveness model shifts—primarily due to the financial burden associated with diagnosing and treating more advanced diseases at later stages. In regions with limited colonoscopy capacity but greater availability of CCE, a higher FIT threshold (e.g., ≤29 µg/g, as suggested in our analysis) may be more appropriate to maximise access and efficiency. Conversely, in settings where conventional colonoscopy is readily available and waiting list pressures are low, a lower FIT threshold (e.g., ≤10 µg/g) for CCE may offer greater cost savings. However, setting the threshold too low could restrict referrals and reduce the overall impact of CCE on relieving endoscopy service demand. Figure 5 showcases our proposed investigation pathway.

Another noteworthy observation was that only 1.6% of patients in our cohort underwent small bowel capsule endoscopy (SBCE) for recurrent iron deficiency anaemia (IDA). This is significantly lower than what has been reported in the literature, where 10–30% of SBCE procedures are performed for the investigation of IDA [41,42]. This discrepancy in our study is likely attributable to service limitations, including restricted access to SBCE and a lack of structured follow-up in both primary and secondary care, particularly the absence of haemoglobin monitoring three months after completion of oral iron therapy. Additionally, as with any retrospective study, the possibility of missing data cannot be excluded. These findings may also reflect a clinical perception that initial OGD and colonoscopy are sufficient for this patient group, potentially leading to the under-recognition of patients with recurrent or unexplained IDA who might benefit from further small bowel evaluation. To date, no studies have evaluated panenteric capsule endoscopy (PCE) as a first-line investigation for patients undergoing small bowel capsule endoscopy (SBCE), particularly in the context of iron deficiency anaemia (IDA). As a result, the true diagnostic yield of PCE in this setting remains uncertain. A revamp of diagnostic pathways is required to enable full utilisation of PCE to assess the clinical utility and cost-effectiveness of using PCE as a primary diagnostic modality in this patient population.

Moreover, other important contributors to CCC—such as incomplete procedures or inadequate bowel preparation in CCE—were not accounted for, meaning the predicted CCC rate is likely underestimated and does not fully reflect real-world PCE outcomes in IDA patients. To accurately assess the clinical value of panenteric capsule endoscopy (PCE) in this setting, a prospective study is needed comparing the diagnostic yield of OGD combined with PCE versus OGD with colonoscopy alone. This should be accompanied by a cost-effectiveness analysis using an appropriate FIT threshold to guide patient selection for the PCE arm. Future advancements may include novel biomarkers, improved imaging technologies, or integrated screening strategies to enhance patient selection for PCE.

### Limitations

This retrospective study has incomplete records on bowel preparation scores and smoking status, both of which were excluded from the analysis. Additionally, selection bias may have occurred, as only patients with complete upper and lower GI investigation reports were included. It is therefore important to conduct a prospective cohort study to validate the effectiveness of FIT thresholds in real-world scenarios, and key covariates should be included to further optimise the CCC rate. The cost–benefit analysis presented was based on a simplified model using published cost estimates for CCE and colonoscopy using regional NHS data from the UK. While this provides a useful comparison, the findings may not be generalisable or transferable to other healthcare systems with different cost structures and healthcare infrastructures. A more comprehensive and locally contextualised economic evaluation would be needed to accurately assess the cost-effectiveness of CCE in broader settings.

## 5. Conclusions

This study indicates that FIT is a suboptimal predictor of CCC in PCE. However, within the iron deficiency anaemia (IDA) cohort, the most cost-effective FIT threshold for minimising the overall service burden appears to lie between 9 and 17 µg/g, based on results from three complementary thresholding methods. These findings may assist clinicians in refining patient selection for PCE, with the optimal FIT cutoff tailored to local factors such as procedure costs, waiting times, and the availability of endoscopy services.

## Figures and Tables

**Table 1:** Baseline patients’ demographics and characteristics.

Number of included patients	1531
Age (mean)	65.4 ± 14.3
Sex	
Male	664 (43.4)
Female	867 (56.6)
Indications	
Asymptomatic IDA	757 (49.4)
FIT positive	250 (16.3)
Dyspepsia	29 (1.9)
Rectal bleeding	49 (3.2)
Dysphagia	24 (1.6)
Change in bowel habit	52 (3.4)
Weight loss	80 (5.2)
Abnormal imaging	18 (1.2)
Abdominal pain	55 (3.6)
Diarrhoea	18 (1.2)
Other *	199 (13)
Smoking	
Smoker	79 (5.2)
Non-smoker	447 (29.2)
Ex-smoker	211 (13.8)
Missing data	794 (51.8)
FIT level (μg/g)	
Median	16
Mean	77.8 ± 122.5
Bowel preparation	
Adequate	641 (41.9)
Poor	40 (2.6)
Missing data	850 (55.5)
Haemoglobin	112.2 ± 19.1
Significant Findings	
Normal	630 (41.1)
Polyp	534 (34.9)
Diverticulosis (Only)	123 (8.0)
CRC	103 (6.7)
Angioectasia	15 (1.0)
IBD	47 (3.1)
Lymphoma	4 (0.3)
Other findings **	75 (4.9)
Number patients with advanced polyps (≥10 mm)	211 (13.8)

**Abbreviations:** CRC, colorectal cancer; FIT, faecal immunochemical test. **Note:** Age, f-Hb mean, and haemoglobin are presented as mean ± standard deviation (SD). Other * refers to indications such as Barrett’s oesophagus, incontinence, melaena, chronic gastrointestinal bleeding, chronic liver disease, and known angiodysplasia. Other findings ** include haemorrhoids only, erythema, non-specific inflammation, solitary rectal ulcer, rectal varices, anal tag, and surgical anastomosis.

## Data Availability

Aggregated additional results are available from the corresponding author upon reasonable request and subject to appropriate justification.

## Supplementary Materials

The following supporting information can be downloaded at: https://www.mdpi.com/article/10.3390/cancers17121951/s1, Table S1: NHS England CCE guidance for polyp surveillance 2020; Table S2: Predefined Clinical Costs and Outcome Assumptions Used in Cost-Benefit Analysis; Table S3: Factors associated with CCE-to-Colonoscopy Conversion (CCC); Table S4: Urgent Colonoscopy Counts, CCC Rates, and Absolute Rate Differences at Identified FIT Jump Thresholds; Figure S1: Distribution curve illustrating participants’ distribution based on FIT levels; Figure S2: ROC curves comparing the predictive performance of FIT alone versus a multivariable model incorporating FIT, haemoglobin (Hb), age, and sex for predicting conversion to conventional colonoscopy (CCC). There was no difference in predictability between the models; Figure S3: Graph illustrating the evolution of statistical significance across the FIT range by plotting *p*-values against FIT levels, highlighting the point at which jumps in CCC rates become statistically significant; Figure S4: Subgroup analysis using multivariable logistic regression to assess differences in CCC rates across FIT-defined subgroups.

## Abbreviations

AI	Artificial Intelligence
BSG	British Society of Gastroenterology
CCC	CCE-to-Colonoscopy Conversion
CCE	Colon Capsule Endoscopy
COVID-19	Coronavirus Disease 2019
CR	Completion Rate
CRC	Colorectal Cancer
CT	Computed Tomography
CTC	CT Colonography
ESGE	European Society of Gastrointestinal Endoscopy
GI	Gastrointestinal
NHS	National Health Service
NPV	Negative Predicted Value
PCE	Panenteric Capsule Endoscopy
