# Supplementary material for: From Stool to Scope: Optimising FIT Thresholds to Guide Future Panenteric Capsule Endoscopy and Reduce Colonoscopy Burden in Iron Deficiency Anaemia
Source: Cancers (Basel). 2025 Jun 11;17(12):1951. doi: 10.3390/cancers17121951 (PMC12191186; doi:10.3390/cancers17121951)
Supplement: Supplementary file 1 [file cancers-17-01951-s001.zip › cancers-3651825-supplementary.pdf]

Table S1: NHS England CCE guidance for polyp surveillance 2020		
Tier level	CCE findings	Follow up Plan
1	1 polyp ≥ 10mm or ≥5 polyps of any size	Proceed directly to therapeutic colonoscopy
2	1 Polyp 6 - 9mm or 3-4 polyps of any size	Deferred therapeutic colonoscopy within 1 year
3	<3 polyps and all <6mm in size	Surveillance colonoscopy at 3 year
4	No polyps	Discharge

Table S2. Predefined Clinical Costs and Outcome Assumptions Used in Cost-Benefit Analysis	
**Classification**	**Value Applied**
True Positive (TP) – correctly referred for colonoscopy	+£747
True Negative (TN) – correctly not referred	+£900
False Positive (FP) – unnecessary colonoscopy	−£153
False Negative (FN) – missed necessary colonoscopy	−£1647
The CCE cost £747 and a colonoscopy cost £900 was referred from the SCOTCAP study[1]	

Table S3 Factors associated with CCE-to-Colonoscopy Conversion (CCC)						
Factors (n=247)	Univariate analysis			Multivariate analysis with LASSO variables		
	Odd ratio	95% CI	p-value	Odd ratio	95% CI	p-value
Age	1.01	1.00-1.02	<0.003**	1.01	1.00-1.02	<0.001***
Sex (M)	1.85	1.45-2.37	<0.001***	1.70	1.30-2.21	0.026*
Haemoglobin	0.99	0.99-1.00	0.06	0.99	0.99-1.00	0.025*
FIT	1.00	1.00-1.01	<0.001***	1.004	1.00-1.01	<0.001***

Table S4 Urgent Colonoscopy Counts, CCC Rates, and Absolute Rate Differences at Identified FIT Jump Thresholds			
FIT Threshold (Jumps)	Number of urgent colonoscopies	CCC rate (%) based on colonic pathologies only	CCC Jump Difference %
7	30	2.0	-
10	71	4.6	2.6
12	85	5.5	0.9
15	92	6.0	0.5
29	128	8.4	2.4
55	176	11.5	3.1
100	212	13.9	2.4
400	341	22.3	8.4

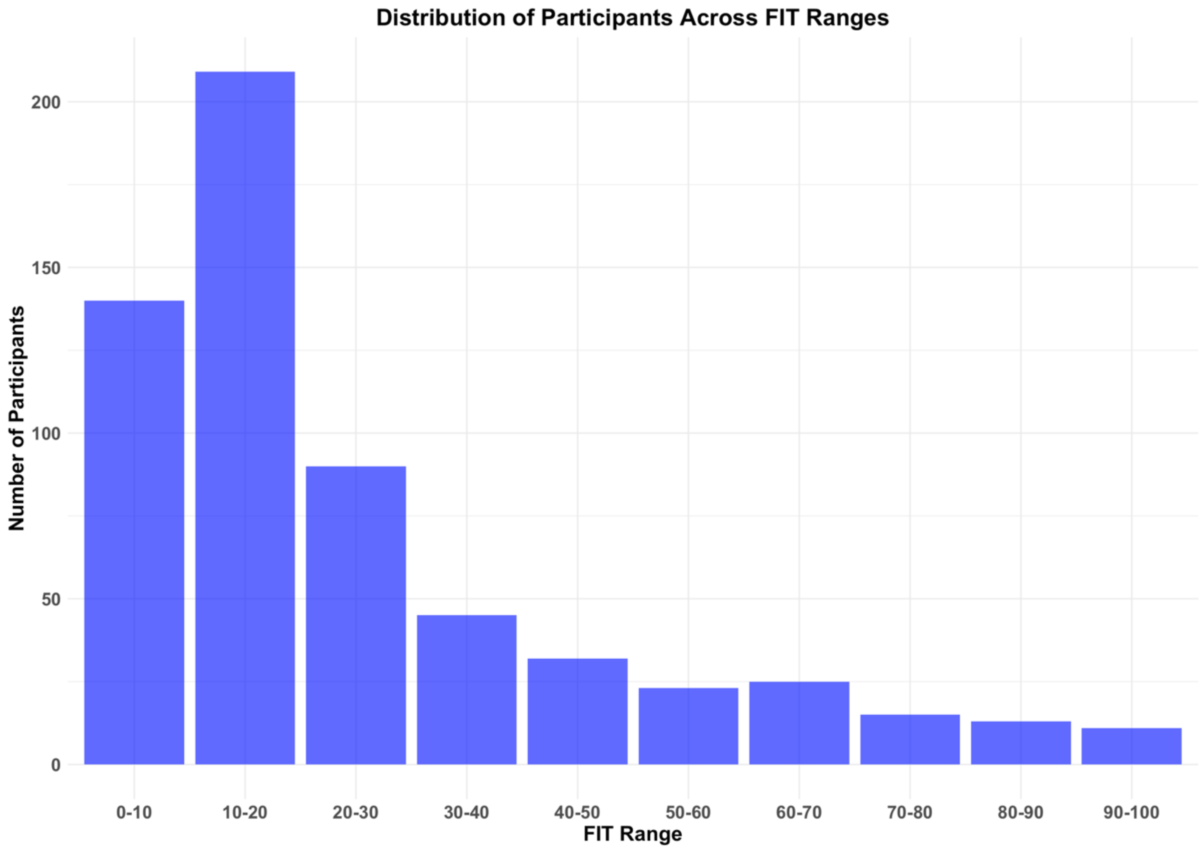

Figure S1. Distribution curve illustrating participants' distribution based on FIT levels.

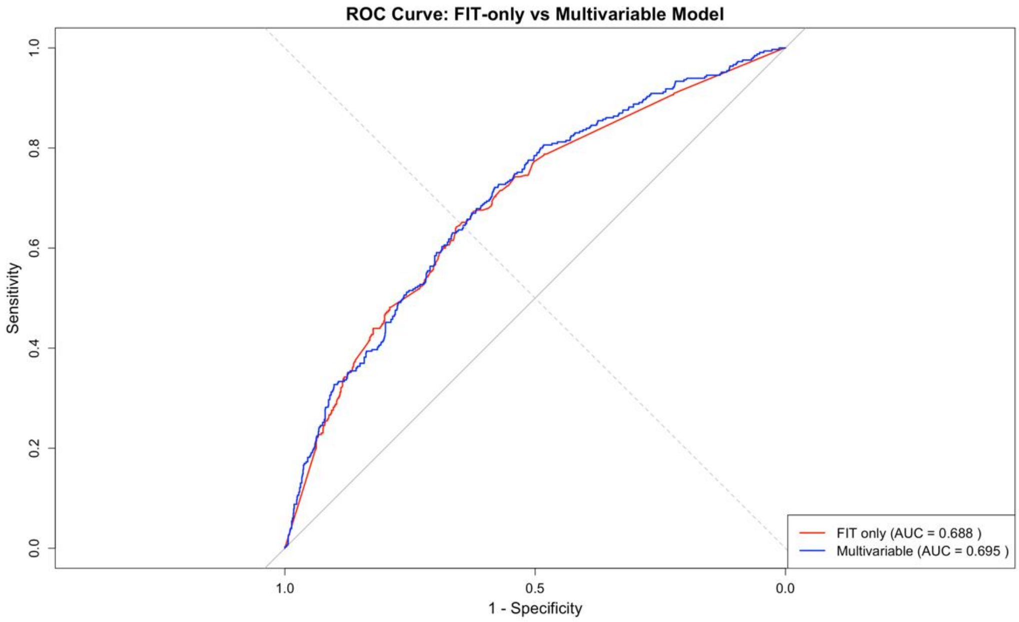

Figure S2. ROC curves comparing the predictive performance of FIT alone versus a multivariable model incorporating FIT, haemoglobin (Hb), age, and sex for predicting conversion to conventional colonoscopy (CCC). There was no difference in predictability between the models.

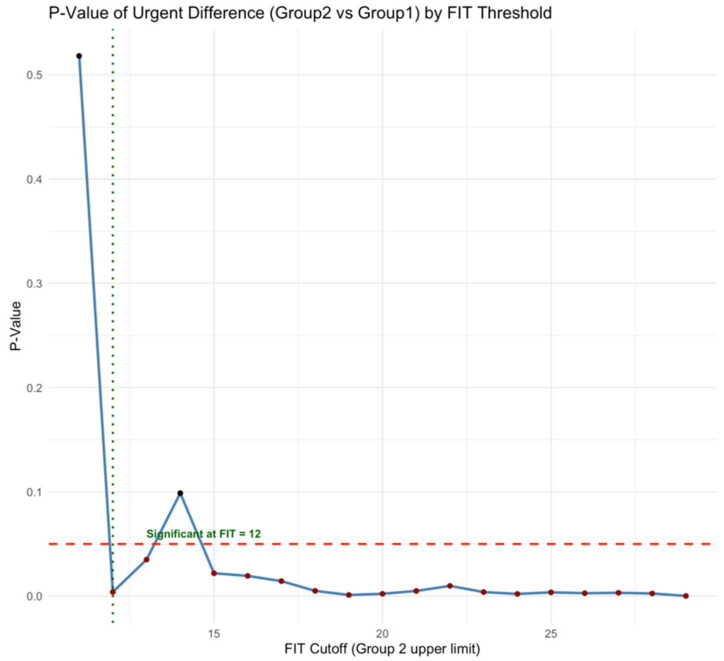

Figure S3. Graph illustrating the evolution of statistical significance across the FIT range by plotting p-values against FIT levels, highlighting the point at which jumps in CCC rates become statistically significant.

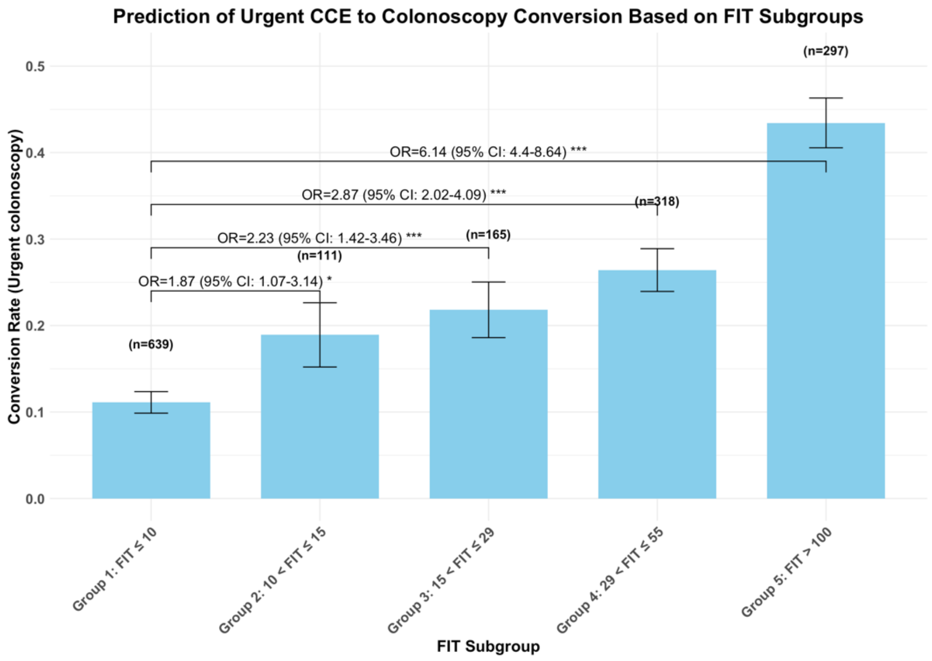

Figure S4. Subgroup analysis using multivariable logistic regression to assess differences in CCC rates across FIT-defined subgroups.

Protocol:

The **CLEAR IDA** Multicentre Audit

***C**CE (Colon Capsule Endoscopy) **L**ed **E**ndoscopic **A**ssessment and **R**eferral using qFIT in*

***I**ron **D**eficiency **A**naemia*

A 12-month retrospective study of the CLEAR IDA Audit.

Audit Protocol

Lead Site: University Hospitals Coventry and Warwickshire NHS Trust

Gastroenterology Research Fellow: Dr Ian Io Lei

Colorectal Nurse Specialists: Ms Nicola O'Connell

Supervised by Prof. Ramesh Arasaradnam

V1.3

15/10/2024

Background:

Iron deficiency anaemia (IDA) is a well-recognized indication for comprehensive gastrointestinal (GI) tract investigation, largely due to concerns about GI blood loss from malignancy. Despite these concerns, the yield of significant findings, particularly gastric or colorectal pathologies, remains relatively low [2-4]. This low detection rate often results in repeated upper GI endoscopies (OGD) and colonoscopies before considering small bowel examinations in cases of recurrent IDA.

The British Society of Gastroenterology guidelines recommend that IDA patients with an inadequate response to iron replacement therapy or recurrent IDA should undergo a small bowel examination as part of their diagnostic workup [5]. This recommendation opens the door for pan-enteric examination as a first-line investigative modality, which could be the most effective approach for this patient group. Such comprehensive examination can be conducted using Colon Capsule Endoscopy (CCE) with the sleep mode deactivated or using the Crohn's capsule.

The Scotcap study revealed that 59% of patients required additional endoscopic investigation (conversion from CCE to conventional endoscopy) after an initial colon capsule endoscopy, which significantly impacted both cost-effectiveness and patient comfort due to the need for repeated bowel preparation (5). To mitigate these issues, incorporating the quantitative fecal immunochemical test (qFIT) as a risk stratification tool for patients with iron deficiency anemia (IDA) could be crucial. By identifying high-risk patients and directing them straight to colonoscopy, the need for re-investigation following CCE could be minimized, improving patient outcomes and optimising resource use. qFIT has already been employed as a risk-stratifying tool in symptomatic patient pathways through the NHS England Pilot project (6, 7). However, the asymptomatic IDA cohort was not included in these indications, and as a result, its findings and the associated colonoscopy conversion rates remain unknown.

Objective:

This study aims to identify factors, including FIT value, that correlate with variables such as pathology and bowel preparation in predicting the conversion from colon capsule endoscopy (CCE) to colonoscopy in this patient group. If FIT is found to be associated with polyp size and number, the next step will be to establish an optimal qFIT threshold that minimises the conversion rate from CCE to colonoscopy in the iron deficiency anaemia cancer pathway which is not part of this audit. The analysis will utilise previous colonoscopy findings within the pathway and apply the NHS England CCE-to-colonoscopy referral criteria, considering the diagnostic comparability between CCE and colonoscopy for polyp detection [6,7].

Methods:

The CLEAR IDA is a multi-centre, retrospective audit encompassing all IDA patients referred through urgent pathways who underwent a qFIT test, OGD, colonoscopy and possible small bowel capsule endoscopy as part of their local referral protocols from 1st September 2023 to 1st September 2024. This audit focuses on identifying pathologies detected during OGD and colonoscopy, with a particular emphasis on the number and size of polyps identified during colonoscopy. The ultimate future goal is to extend these findings to colon capsule endoscopy (CCE) to identify the factors influencing colonoscopy conversion and determine the optimal qFIT threshold that minimises conversion rates which is not part of this audit. This approach aims to reduce delays in the further investigation and management of malignancies.

Data will be primarily sourced from primary care referral letters, biochemistry reports, and endoscopy reports. By analysing the number and size of polyps found and applying the NHS England CCE criteria, we will hypothetically evaluate whether these patients would have met

the criteria for CCE to colonoscopy conversion, thus providing insights into the potential effectiveness of CCE as a first-line investigation in this pathway.
Patients will be identified at local sites using their endoscopy referral systems, focusing on those referred for iron deficiency anaemia. No direct patient contact will occur during this audit. Each site will be responsible for registering the audit according to local guidelines. Data will be captured using an offline spreadsheet specifically designed for this purpose. Additionally, national data opt-out screening will be conducted by the local teams, with support from their audit departments, to exclude participants who have opted out of national audit data usage.
This observational audit aims to study the performance of the FIT test (standard practice) in identifying significant pathologies in patients with IDA.

Participant Inclusion Criteria:

1) Adults aged 18 years or older.
2) Confirmed iron deficiency with or without anaemia, including microcytic anaemia or low ferritin levels without anaemia.
3) Underwent FIT testing, OGD, and colonoscopy, with or without small bowel capsule endoscopy.

Statistical Analysis Plan:

A power calculation was performed to determine the required sample size using the following parameters:

- Effect size – 0.8
- Alpha – 0.05
- Power – 0.9
- Standard deviation of qFIT value: 117
- Delta - 20

The calculated sample size is 1,441 participants. The study analysis will include logistic regression to assess whether factors such as qFIT, sex, age, and haemoglobin levels correlate with the number of polyps, the presence of advanced polyps (size > 10 mm), the detection of colorectal cancer and colonoscopy conversion.

Reference:

[1] Scotland HI. colon capsule endoscopy for detection of colorectal polyps and cancer. Innovative Medical Technology Overview. Feb 2024.
[2] Cilona A, Zullo A, Hassan C, et al. Is faecal-immunochemical test useful in patients with iron deficiency anaemia and without overt bleeding? Dig Liver Dis. 2011 Dec;43(12):1022-4.
[3] American Gastroenterological Association medical position statement: evaluation and management of occult and obscure gastrointestinal bleeding. Gastroenterology. 2000 Jan;118(1):197-201.
[4] James MW, Chen CM, Goddard WP, et al. Risk factors for gastrointestinal malignancy in patients with iron-deficiency anaemia. Eur J Gastroenterol Hepatol. 2005 Nov;17(11):1197-203.

[5] Snook J, Bhala N, Beales ILP, et al. British Society of Gastroenterology guidelines for the management of iron deficiency anaemia in adults. Gut. 2021 Nov;70(11):2030-2051.
[6] Alihosseini S, Aryankhesal A, Sabermahani A. Second-generation colon capsule endoscopy for detection of colorectal polyps: A meta-analysis. Med J Islam Repub Iran. 2020;34:81.
[7] Mollers T, Schwab M, Gildein L, et al. Second-generation colon capsule endoscopy for detection of colorectal polyps: Systematic review and meta-analysis of clinical trials. Endosc Int Open. 2021 Apr;9(4):E562-E571.
